# Ostracism

**Published:** 1887-06

**Authors:** P. P. Wells

**Affiliations:** Brooklyn, N. Y.


### HOMŒOPATHIC PHYSICIAN,

A MONTHLY JOURNAL OF MEDICAL SCIENCE.

"If our school ever gives up the strict inductive method of Hahnemann, we are lost, and deserve only to be mentioned as a caricature in the history of medicine."—CONSTANTINE HERING.

Vol. VII. JUNE, 1887. No. 6.

### OSTRACISM.

P. P. WELLS, M. D., BROOKLYN, N. Y.

"We have been ostracized because we would teach the truth, the whole truth, and nothing but the truth."—*Dean of the Faculty, in his Commencement Speech.*

Ostracized! Where? when? and by whom? If this be true of this College faculty (for it could only be of them he spoke), it is not a little singular we never heard of it before. But this is not so surprising as the cause given for this grievous treatment. We have never heard that this faculty has been accused, or even suspected of teaching "the whole truth"! We have heard them accused of omitting to teach the very truth for which their College had been created, that therein it might be taught, and this because this truth could not be elsewhere taught. [So said this Dean in a previous commencement speech.] The chairs occupied by this faculty were created, and their present incumbents appointed to them, that from these this truth might assuredly be taught, and yet they have been publicly accused of neglecting to teach it! They have been urged by "our great representative body" and by an ex-member of the faculty to cease this neglect, and begin, now, after these many years of sin, to "teach pure Homœopathy"—the only *raison d'être* of this College and of its professors as such. They have been thus accused before the public, *not* for teaching "the whole truth," but for omission in their teaching of the only truth in the interests of which they were supposed to have existence. We have but recently so accused them ourselves, and given our reasons for so doing, and up to this time this College has not replied to the urgency of our "great representative body," nor to that of its ex-member (approved by this Dean, who now pretends ostracism of his College because they have taught it). Till this last utterance of this too-much-talking Dean there has been no pretense from this faculty that when so accused they were suffering any injustice. They have not denied the truth of the accusation, because truthfully they could not. If they could do this, why not, when subjected to the ostracism complained of, present the fact in bar of the unjust judgment? If they were to attempt any denial of the truth of this charged neglect, the great crowd of their untaught and ignorant graduates would stand, living witness, to the falsehood and impudence of this denial, which for enormity would stand without a parallel!

And, then, the Dean says they "have been ostracized"—this College of his—because they would teach "nothing but the truth"! Surely a greater departure from "truth" than this, this careless-talking Dean could hardly have been capable of! It admits, so far as we can see, of only one explanation—he did not know what was taught in his College. We would kindly refer him, for examples of teaching wholly subversive of the "truth" he and his co-professors were appointed to teach, to THE HOMŒOPATHIC PHYSICIAN for May, 1887, where he will find the false of old physic substituted for this truth he claims to have been taught, and that nothing else has been taught with it. And the only excuse we can see for this utterance of the Dean, and this is a poor one, is his desire, in the excitement of talking, to give to others, who did not know, a well-sounding sentence which should cover much ground. And for this purpose what could be better than this—"The truth, the whole truth, and nothing but the truth"? What though, in this one little sentence of eighteen words, there be no less than four absolute falsehoods, this audience don't know this, and then, given with a bold face, it sounds well, you know.

We say there are no less than *four* falsehoods, and here they are:

First. It is not true that this Dean or his College have been ostracized at all.

Second. It is not true that they have taught the "truth" in the interests of which this College was created.

Third. It is not true they have taught the "whole truth," this most important of all, that of specific therapeutics having been confessedly omitted, as is proved by the exhortation of their ex-Professor, indorsed by this Dean, that they should now begin to teach this.

Fourth. It is not true that the faculty of this College have taught "nothing but the truth." They have taught that the clumsy and destructive results of old physic for the cure of gonorrhœa are superior to the true specific remedy for this disease, and also a similar superiority of similar resorts to the specific remedy for uterine hemorrhage; and these teachings are not truths, but *lies!*

We can imagine only one origin of this idea of ostracism of this College, as it has been banished to or from nowhere, and that is, in an imagination sorely pressed by a consciousness of ill-desert for so protracted a course of abuse of public confidence, by so long-continued official neglect. Conscience may have so long asserted the desert of ostracism, that its voice has come at last to have the force of so impressing the imagination of this Dean that he has become incapable of distinguishing it from the fact he complains of. He knows his College *ought* to be ostracized from the confidence of all good men, by reason of neglect to teach law, and the goadings of conscience therefore have been so hard on him that he has come at last to believe (if he did believe) that his College had received its just deserts.

Now, what resort for relief of this supposed ostracism could be more promising than an appeal to the sympathies of his audience by posing before it as a suffering martyr? And then a martyr for the "*truth*," which this College had only neglected or abused! Is the pretense so gross as to leave the resort only a bald absurdity? But then this audience knew nothing of the sin of this College, and its Dean knew they knew nothing of it, and he ventured the experiment which has placed him in danger of banishment from the confidence of truth-loving men.

