# Correction

**Published:** 1887-07

**Authors:** 


Correction.—June number, page 183, second line from the top, for "results" read *resorts.*

